# Circadian rhythms have significant effects on leaf-to-canopy scale gas exchange under field conditions

**DOI:** 10.1186/s13742-016-0149-y

**Published:** 2016-10-20

**Authors:** Víctor Resco de Dios, Arthur Gessler, Juan Pedro Ferrio, Josu G. Alday, Michael Bahn, Jorge del Castillo, Sébastien Devidal, Sonia García-Muñoz, Zachary Kayler, Damien Landais, Paula Martín-Gómez, Alexandru Milcu, Clément Piel, Karin Pirhofer-Walzl, Olivier Ravel, Serajis Salekin, David T. Tissue, Mark G. Tjoelker, Jordi Voltas, Jacques Roy

**Affiliations:** 1 Department of Crop and Forest Sciences Agrotecnico Center, Universitat de Lleida, 25198 Lleida, Spain; 2 Swiss Federal Institute for Forest, Snow and Landscape Research, Long-term Forest Ecosystem Research, 8903 Birmensdorf, Switzerland; 3 Institute for Landscape Biogeochemistry, Leibniz Centre for Agricultural Landscape Research, 15374 Müncheberg, Germany; 4 Departamento de Botánica, Facultad de Ciencias Naturales y Oceanográficas, Universidad de Concepción, Casilla 160-C, Concepción, Chile; 5 School of Environmental Sciences, University of Liverpool, Liverpool, L69 3GP UK; 6 Institute of Ecology, University of Innsbruck, 6020 Innsbruck, Austria; 7 Ecotron Européen de Montpellier, UPS 3248, Centre National de la Recherche Scientifique, Campus Baillarguet, 34980 Montferrier-sur-Lez, France; 8 Instituto Madrileño de Investigación y Desarrollo Rural, Agrario y Alimentario, Finca ‘El Encín’, 28800 Alcalá de Henares, Madrid Spain; 9 USDA Forest Service, Northern Research Station, Lawrence Livermore National Laboratory, Livermore, California, 94550 USA; 10 Centre National de la Recherche Scientifique, Centre d’Ecologie Fonctionnelle et Evolutive, UMR 5175, Université de Montpellier, Université Paul Valéry, École Pratique des Hautes Études, F-34293 Montpellier Cedex 5, France; 11 Institut für Biologie, Plant Ecology, Freie Universität Berlin, D-14195 Berlin, Germany; 12 Berlin-Brandenburg Institute of Advanced Biodiversity Research, D-14195 Berlin, Germany; 13 Erasmus Mundus Master on Mediterranean Forestry and Natural Resources Management, Universitat de Lleida, 25198 Lleida, Spain; 14 School of Forestry, College of Engineering, University of Canterbury, 8140 Christchurch, New Zealand; 15 Hawkesbury Institute for the Environment, Western Sydney University, Richmond, NSW 2753 Australia

**Keywords:** Circadian clock, Ecological memory, Net ecosystem exchange, Scaling, Stomatal conductance models, Photosynthesis, Transpiration

## Abstract

**Background:**

Molecular clocks drive oscillations in leaf photosynthesis, stomatal conductance, and other cell and leaf-level processes over ~24 h under controlled laboratory conditions. The influence of such circadian regulation over whole-canopy fluxes remains uncertain; diurnal CO_2_ and H_2_O vapor flux dynamics in the field are currently interpreted as resulting almost exclusively from direct physiological responses to variations in light, temperature and other environmental factors. We tested whether circadian regulation would affect plant and canopy gas exchange at the Montpellier European Ecotron. Canopy and leaf-level fluxes were constantly monitored under field-like environmental conditions, and under constant environmental conditions (no variation in temperature, radiation, or other environmental cues).

**Results:**

We show direct experimental evidence at canopy scales of the circadian regulation of daytime gas exchange: 20–79 % of the daily variation range in CO_2_ and H_2_O fluxes occurred under circadian entrainment in canopies of an annual herb (bean) and of a perennial shrub (cotton). We also observed that considering circadian regulation improved performance by 8–17 % in commonly used stomatal conductance models.

**Conclusions:**

Our results show that circadian controls affect diurnal CO_2_ and H_2_O flux patterns in entire canopies in field-like conditions, and its consideration significantly improves model performance. Circadian controls act as a ‘memory’ of the past conditions experienced by the plant, which synchronizes metabolism across entire plant canopies.

**Electronic supplementary material:**

The online version of this article (doi:10.1186/s13742-016-0149-y) contains supplementary material, which is available to authorized users.

## Background

Terrestrial ecosystems play a major role in global carbon and water cycles. Current estimates suggest that ~30 % of fossil fuel emissions are sequestered by land [1], and that ~60 % of annual precipitation is returned to the atmosphere through evapotranspiration; a flux largely dominated by transpiration [2]. There is a long tradition within Earth Sciences research on deciphering the mechanisms underlying diurnal variations in photosynthesis and transpiration [3–6]. This research has mostly focused on direct physiological responses to the environment; i.e., understanding how photosynthetic machinery and stomatal function respond and react to changes in radiation, temperature, vapor pressure deficit, and other environmental drivers.

A smaller body of research has sought to disentangle whether, apart from direct responses to exogenous factors, endogenous processes could also play a role [7]. It has been documented, for instance, that for a given level of water potential and abscisic acid (ABA) concentration, stomatal conductance is higher in the morning than it is in the afternoon [8]. The circadian clock controls this phenomenon [8]; an endogenous timer of plant metabolism that controls the temporal pattern of transcription in photosynthesis, stomatal opening, and other physiological processes [9]. Additional processes create endogenous flux variation, such as carbohydrate accumulation or apparent feed-forward stomatal responses [3, 10], but only the circadian clock shows a 24 h oscillation.

Ample evidence indicates the circadian regulation of leaf photosynthesis and conductance under controlled environmental conditions [11, 12]. However, processes regulating fluxes at the leaf scale will not necessarily also regulate fluxes at the canopy or ecosystem scales – not all processes relevant at one scale also act upon the ‘next’ scale [13]. Unfortunately, research on the circadian regulation of photosynthesis and transpiration within field settings has received limited attention. Those studies mentioning circadian regulation often consider it to be a negligible driver at canopy or ecosystem scales [14, 15], although there are a few notable exceptions [16, 17].

Direct assessments of the circadian regulation of gas exchange over entire plant canopies are complicated because of logistical constraints (e.g., the need to control all sources of environmental variation, including radiation, over entire ecosystems). Instead, the scarce attempts to infer circadian regulation of carbon assimilation in the field at canopy or ecosystem scales have been achieved indirectly by filtering flux tower data, and have obtained circumstantial support for a significant effect of circadian regulation over net ecosystem CO_2_ exchange in the field [18, 19]. Others, working with nocturnal transpiration, have additionally documented that circadian regulation, over nocturnal stomatal conductance, affects the transpiration stream in whole trees [20] or even entire plant canopies [21].

Understanding whether or not circadian regulation in diurnal gas exchange is important at the leaf-level to the canopy-level scales requires an understanding of the potential implications of the circadian clock as a driver of ecosystem flux dynamics; there is some expectation that a dilution of circadian effects will occur as we move up in scale. Circadian rhythms are entrained by environmental cues of light and temperature; therefore, at the canopy scale, different leaves will experience different light and temperature cues. Hence, we could observe uncoupled circadian rhythms in different leaves within and across plants, potentially diluting any circadian effects at canopy scales.

Moreover, along with the capacity to detect circadian rhythms at canopy scales, additional studies are required that include circadian regulation in gas exchange modeling. To our knowledge, the only study that has so far tested the relevance of circadian rhythmicity for modeling gas exchange in the field concluded that, “circadian rhythms have insignificant effects on plant gas exchange under field conditions” [22]. This was a pioneer study that, for the first time, took research on circadian rhythms outside of lab settings and worked with a non-model species from wetland and understory environments (*Saururus cernuus* L). The researchers [22] measured leaf-level fluxes under “constant environmental conditions” (i.e., when temperature, radiation, and other environmental drivers do not change over time) and documented a 24-h oscillation in gas exchange within growth chambers, consistent with circadian regulation of gas exchange. Subsequently, they tested whether or not circadian regulation would be significant in the field by adding a sinusoidal variation to a biochemical model of gas exchange, and then comparing modeled output with field-measured data. Under these field conditions, they observed that model goodness-of-fit increased by only 1 %; therefore it was concluded that circadian regulation of gas exchange in the field was insignificant. These negative results may partly explain the lack of interest in the circadian regulation of gas exchange in the field. However, this study was focused on photosynthesis and, to our knowledge, we are unaware of attempts to include circadian regulation in stomatal conductance models.

Here, we use large macrocosms within an experimental Ecotron [23] to monitor leaf and canopy gas exchange under field-like and constant environmental conditions in bean (*Phaseolus vulgaris*) and cotton (*Gossypium hirsutum*) canopies. We tested i) whether circadian regulation in photosynthesis and daytime stomatal conductance scales up from leaf-level to canopy-level; and ii) whether adding a circadian oscillator into well-known stomatal models would significantly increase model fit with observed data.

## Data description

Diurnal variation in canopy and ecosystem fluxes is largely attributed to the direct environmental effects of photosynthetically active radiation (PAR), air temperature (*T*_air_), and vapor pressure deficit (VPD) on physiological processes [3–6]. Currently lacking in the literature is an experiment acting as a ‘control’, whereby fluxes over entire plant canopies or ecosystems are measured under constant environmental conditions. Addressing this deficit, we assessed assimilation and transpiration in entire canopies under a fluctuating environment, and compared this with constant environmental conditions, to understand what diurnal range in canopy CO_2_ and H_2_O fluxes can be recreated, fully independently of environmental change. We hypothesized that if data revealed a temporal pattern under constant environmental conditions, also following a sinusoidal oscillation and with a ~24 h cycle, this would indicate circadian regulation at canopy scales. Additionally, we collected leaf-level gas exchange data to clarify whether considering circadian regulation would improve the performance of stomatal conductance models.

The experiment was performed at the Macrocosms platform of the Montpellier European Ecotron, Centre National de la Recherche Scientifique (CNRS, France). We used 12 outdoor macrocosms (six planted with bean and six with cotton) where the main abiotic drivers (air temperature, humidity, and CO_2_ concentration) were automatically controlled. Each macrocosm was designed as an open gas exchange system to continuously measure CO_2_ net ecosystem exchange by measuring air flow at the inlet of each dome (using a thermal mass flowmeter; Sensyflow iG, ABB, Zurich, Switzerland) and by sequentially (every 12 min) measuring the CO_2_ concentration at each inlet and outlet using a multiplexer system coupled with two LI-7000 CO_2_/H_2_O analyzers (LI-COR Biosciences, Lincoln, NE, USA). Substantial internal air-mixing within the dome (two volumes per min) reduced the canopy boundary layer resistance and minimized the CO_2_ concentration gradients within the dome. Belowground fluxes were prevented from mixing with canopy air by covering the soil with a plastic sheet during the entire experimental period. A slight atmospheric over-pressure (5–10 Pa) applied in the domes forced some air to flow below the plastic sheet (through the slits made for the plant stems). This air flow flushed away the CO_2_ respired by the soil and minimized potential mixing of soil respiration fluxes with aboveground fluxes. Indeed, we observed negligible CO_2_ flux at the onset of the experiment (immediately after seed germination, when there was no significant carbon assimilation), indicating no effect of soil CO_2_ efflux on the canopy above the plastic sheet. Transpiration was measured continuously by weighing lysimeters with four shear beam load cells per lysimeter (CMI-C3 Precia-Molen, Privas, France), and calculated from the slope of the temporal changes in mass using a generalized additive model with automated smoothness selection [24]. Further details on Ecotron measurements are described elsewhere [23, 25].

For each crop, three macrocosms were dedicated to leaf-level measurements (researchers entered periodically) and the remaining three ‘undisturbed’ macrocosms were dedicated to continuous canopy gas exchange measurements. At the end of each experiment, leaf area was measured in five randomly selected plants from each macrocosm, using a leaf area meter (LI-3100C, LI-COR Biosciences, Lincoln, NE, USA). Leaf area index (LAI) was estimated by multiplying average leaf area by the number of individuals in a macrocosm, divided by ground area. LAI was 7.5 m^2^ m^−2^ in bean and 4.5 m^2^ m^−2^ in cotton. Though this was much higher than seen in field settings, a higher LAI was desirable because it leads to a higher proportion of shaded leaves, which in turn, should decrease the relative effect of circadian regulation over canopy scales. This will be discussed in detail later.

Leaf gas exchange was measured using a portable photosynthesis system (LI-6400XT, Li-Cor, Lincoln, Nebraska, USA), after setting the leaf cuvette to the same environmental conditions as the macrocosms. We conducted spot gas exchange measurements every 4 h in three leaves from the upper light-exposed part of the canopy within each macrocosm, and average values for each of the three macrocosms per species were used in subsequent analyses. Different leaves from different individuals were measured during each round of measurement. Leaf temperature was independently measured at the time of gas exchange measurements using an infrared thermometer (MS LT, Optris GmbH, Berlin, Germany). No significant difference with air temperature was recorded by the *T*_air_ probe (PC33, Mitchell Instrument SAS, Lyon, France) (intercept = −4.3 ± 4.5 [mean ± 95 % CI]; slope = 1.15 ± 0.17; R^2^ = 0.89).

## Analyses

*Question 1: Does circadian regulation scale up to affect whole canopy fluxes?*

**Fig. 1:**
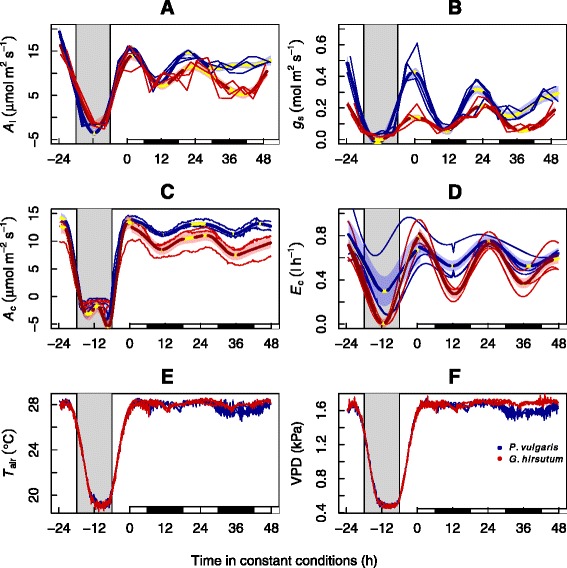
Circadian regulation of leaf and canopy-scale CO_2_ and H_2_O fluxes. We grew cotton (*Gossypium hirsutum*) and bean (*Phaseolus vulgaris*) in experimental macrocosms at the Montpellier European Ecotron. During experimental conditions, temperature (*T*_air_, **e**) and vapor pressure deficit (VPD, **f**) mimicked the environmental conditions of an average August day in Montpellier, with 500 μmol m^−2^ s^−1^ photosynthetically active respiration (PAR) (first 24 h shown) remaining constant for the following 48 h starting at solar noon. Grey or white background indicate when PAR was at or above 0 μmol m^−2^ s^−1^ respectively. The white and black rectangles at the base indicate the subjective day (when it would have been daytime during entrainment) and subjective night, respectively, under constant conditions. Thin lines represent measured values at each of three replicate macrocosms, and thick lines (and shaded error intervals) indicate the prediction (and SE) of generalized additive mixed model (GAMM) fitting separately for each species (some lines may overlap). Portions of the thick line in yellow indicate lack of statistical variation in the slope. Significant variation (GAMM best-fit line portions not yellow) in leaf (**a**) and canopy (**c**) carbon assimilation (*A*_l_ and *A*_c_, respectively), stomatal conductance (*g*_s_, **b**) and canopy transpiration (*E*_c_, **d**) prevailed for all fluxes and processes, at least in the first 24 h under constant conditions. This can be fully attributed to circadian action. Clock regulation is plastic and may relax after prolonged exposures to constant conditions [56]. Negative dark-time values of *A*_l_/*g*_s_ and *A*_c_/*E*_c_ were cropped as they lack biological meaning

We tested whether leaf circadian regulation scaled up to affect whole ecosystem CO_2_ and H_2_O fluxes by examining leaf carbon assimilation (*A*_l_) and stomatal conductance (*g*_s_), in addition to canopy carbon assimilation (*A*_c_) and transpiration (*E*_c_) under ‘constant’ and ‘changing’ environmental conditions. Canopies were originally entrained (‘changing’ conditions) by mimicking the temporal patterns in *T*_air_ (28/19 **°**C, max/min) and VPD (1.7/0.5 kPa) of an average sunny August day in Montpellier (Fig. 1). Photoperiod was set to 12 h of darkness and 12 h of light during entrainment, and a maximum PAR of 500 μmol m^−2^ s^−1^ at canopy height, was provided by the plasma lamps. We acknowledge that this radiation level is substantially lower than that usually experienced on a sunny day in Montpellier, but at the time of the experiment, we were not aware of any other facilities that provided environmental control and automated flux measurements at canopy scales under a higher radiation. After a 5-day entrainment period, we maintained constant PAR, *T*_air_ and VPD for 48 h, starting at solar noon (‘constant’ conditions). We observed continuous temporal variation in leaf-level and integrated canopy CO_2_ (*A*) and H_2_O (*E*) fluxes between 20 % and 79 % of the range observed during entrainment (details following; also see Fig. 1 and Table 1). Temporal variations of *A* and *E* at the leaf and canopy-levels under a constant environment showed a period of ~24 h, consistent with the circadian regulation of leaf photosynthesis (*A*_l_) and stomatal conductance (*g*_s_).

**Table 1:** Quantification of the circadian-driven range in variation of diurnal gas exchange

Process	Species	Scale	Variation during entrainment			Variation during constant conditions			% clock-driven variation
			Max (SE)	Min	Max-Min	Max (SE)	Min (SE)	Max-Min	
Carbon assimilation	*P. vulgaris*	Leaf (μmol m^−2^ s^−1^)	19.30 (0.97)	0	19.30	15.67 (0.66)	7.79 (0.63)	7.88	40.83
		Ecosystem (μmol m^−2^ s^−1^)	14.21 (0.37)	0	14.21	13.92 (0.32)	11.12 (0.30)	2.79	19.67
	*G. hirsutum*	Leaf (μmol m^−2^ s^−1^)	16.32 (1.42)	0	16.32	14.00 (0.80)	5.13 (0.84)	8.87	54.35
		Ecosystem (μmol m^−2^ s^−1^)	13.38 (1.11)	0	13.38	12.51 (0.91)	7.48 (0.90)	5.03	37.63
Water fluxes	*P. vulgaris*	Leaf (conductance, mol m^−2^ s^−1^)	0.48 (0.04)	0	0.48	0.43 (0.03)	0.05 (0.03)	0.38	79.17
		Ecosystem (l h^−1^)	0.40 (0.07)	0	0.40	0.37 (0.03)	0.25 (0.03)	0.12	28.39
	*G. hirsutum*	Leaf (conductance, mol m^−2^ s^−1^)	0.22 (0.02)	0	0.22	0.21 (0.01)	0.05 (0.01)	0.16	72.73
		Ecosystem (l h^−1^)	0.39 (0.04)	0	0.39	0.39 (0.03)	0.14 (0.03)	0.25	64.55

The variation in fluxes attributable to the clock in Fig. 1 was derived from the ratio between the range (maximum value predicted by generalized additive mixed model (GAMM) analysis minus minimum GAMM predicted value) in each flux while keeping environmental conditions constant (the last 48 h shown in Fig. 1), divided by the range during the entrainment phase (first 24 h in Fig. 1). Although nocturnal stomatal conductance and transpiration were always above 0 during entrainment, even during dark periods, we forced their minimum to be zero for this calculation. This increased the magnitude of the variation during entrainment, thus leading to under-estimations of the % variation attributable to the clock. Nocturnal carbon assimilation was also fixed at 0, because no C assimilation occurs in the dark

We examined statistical significance of these temporal patterns, and calculated the diurnal variation range under constant and changing environmental conditions, using a generalized additive mixed model (GAMM) fitted with automated smoothness selection [24] in the R software environment (*mgcv* library in R 3.1.2, R Foundation for Statistical Computing, Vienna, Austria), including macrocosms as a random factor. This approach makes no *a priori* assumption about the functional relationship between variables. We accounted for temporal autocorrelation in the residuals by adding a first-order autoregressive process structure (*nlme* library [26]). Significant temporal variation in the GAMM best-fit line was analyzed after computation of the first derivative (the slope, or rate of change) with the finite differences method. We also computed standard errors (SE) and a 95 % point-wise confidence interval for the first derivative. The trend was subsequently deemed significant when the derivative confidence interval was bounded away from zero at the 95 % level (for full details on this method see [27]). Non-significant periods, reflecting lack of local statistically significant trending, are illustrated on the figures by the yellow line portions, and significant differences occur elsewhere. The magnitude of the range in variation driven by the circadian clock (Table 1) was calculated using GAMM maximum and minimum predicted values.

In terms of the magnitude of the oscillation, there were some differences across species but, overall, similar patterns were observed (Fig. 1, Table 1). Circadian regulation was diluted as we moved up in scale. For instance, the magnitude of the clock-driven variation was 41/54 % of the total diurnal oscillation during entrainment for *A*_l_ (bean/cotton), but 20/38 % for *A*_c_. Similarly, while *g*_s_ varied by 72/79 % under constant conditions, variation in *E*_c_ was 28/64 %. However, despite this dilution, we always observed a significant self-sustained 24-h oscillation in *A*_c_ as well as in *E*_c_.

It could be argued that calculating the magnitude of circadian regulation tends to overestimate its importance because it is based upon a 24-h cycle, whereas in reality, no *A*_c_ occurs during the night, and *E*_c_ will be lower during a normal night (when it is dark) than in the ‘subjective’ night (the period under constant conditions when it would have been night-time during entrainment). Hence, we re-calculated the magnitude of the oscillation in *A*_c_ and *E*_c_ only during the 12 h of the subjective day under constant conditions and observed that it was 15.4 % and 24.0 %, respectively, for bean, and 29.75 % and 37.7 %, respectively, for cotton.

*Question 2: Does adding a circadian oscillator improve the performance of stomatal models?*

Having established the importance of circadian control over canopy-level processes, we aimed to test whether adding a circadian oscillator into well-known stomatal models would significantly increase model fit. The models used [28–30] have two common fitting parameters, *g*_0_ (minimal conductance, or the intercept of the model) and *g*_1_ (the slope relating *g*_s_ to *A*_l_ and environmental variables). Models were run with and without *g*_0_, as the interpretation of minimal conductance remains elusive [28]. We observed changing *A*_l_ and *g*_s_, so circadian oscillations were added to modify the values of *g*_1_ over time (Eq. 1):

1 $$ {g}_1={g}_{1\mathrm{m}}+{g}_{1\mathrm{a}} \sin\ \left({g}_{1\mathrm{f}}2\pi t/24 + {g}_{1\mathrm{p}}\right) $$

where subscripts *m*, *a*, *f* and *p* indicate the mean *g*_1_ value, the amplitude, frequency and phase of the rhythm, respectively, and *t* is time in hours (since experiment onset). That is, we studied the clock effect on *g*_s_ model predictions by comparing the original model formulations [28–30] before (without circadian oscillator), and after (with circadian oscillators), replacing *g*_1_ in the original formulations by Equation 1. We derived *g*_1m_ for models including a circadian oscillator from the estimate of *g*_1_ in the corresponding models without a circadian oscillator, and the frequency (*g*_1f_) was additionally fixed at 24 h (*g*_1f_ = 1).

Three different model runs were conducted for each of the three different stomatal conductance models. First, each *g*_s_ model was calibrated and validated with the entire leaf-level data set (Fig. 1). Second, we calibrated each model under changing diurnal conditions of PAR, *T*_air_ and VPD (first 24 h in Fig. 1) and validated it with data under constant PAR, *T*_air_ and VPD conditions (last 48 h in Fig. 1). Third, we calibrated each model under constant PAR, *T*_air_ and VPD conditions, and validated it with data under changing PAR, *T*_air_ and VPD. Given the distinctly different patterns of environmental conditions during the changing and constant phases, the last two model runs were included to represent changes in model fit under ‘novel’ environmental conditions. Importantly, the third model run was comparable with previous studies on the importance of circadian regulation for modeling in the field [22] because it used data collected under constant environmental conditions to infer the effect on *g*_s_ over changing environmental conditions.

The models were fitted independently for each species, but observed and predicted values were combined for validation. We calculated R^2^ from the regression between observed versus predicted values, and Akaike information criterion (AIC) was obtained as (Eq. 2):

2 $$ \mathrm{A}\mathrm{I}\mathrm{C} = -2L\left(\mathrm{M}\mathrm{L}\mathrm{E}\right) + 2p $$

where *L*(MLE) is the likelihood function evaluated at the maximum likelihood estimates, and *p* is the number of parameters. AIC reduction (∆AIC) for each model was calculated from the difference to the smallest AIC. The weight of each model (*w*_i_), which indicates the conditional probability of each model, was calculated from the ratio between the relative likelihood of a model (calculated as e^(−0.5 ∆AIC)^) to the sum of the relative likelihood of all the models [31, 32].

Depending on the combination of data sets, we observed that either variation from the models originally proposed by [28] or by [29] performed the best (Table 2). However, regardless of the data set, the best model was always one that included a circadian oscillator in the slope (Table 2). When using the entire data set for calibration and validation, R^2^ of the predicted-versus-observed relationship increased by 17 % (from 0.66 to 0.83) when adding a circadian oscillator. When using the data set under changing conditions for calibration, and the data set under constant conditions for validation, the R^2^ increased by 12 %, from 0.60 to 0.72, after including a circadian oscillator. Finally, when using the data set under constant conditions for calibration, and the data set under changing conditions for validation, which would have been comparable to previous experiments [22], we observed that the R^2^ of the predicted-versus-observed relationship increased by 8 %, from 0.74 to 0.82, after including a circadian oscillator. It is important to note that all stomatal models include photosynthesis as a driver, which likely explains the high goodness of fit even when the model is parameterized using only data collected under constant conditions (see Additional file 1). Despite the higher number of parameters, the model with a circadian oscillator also consistently exhibited smaller AIC across all combinations of calibration/validation data sets. This result indicates that current stomatal models show a high predictive power, but that inclusion of a circadian oscillator significantly improves their performance.

**Table 2:**
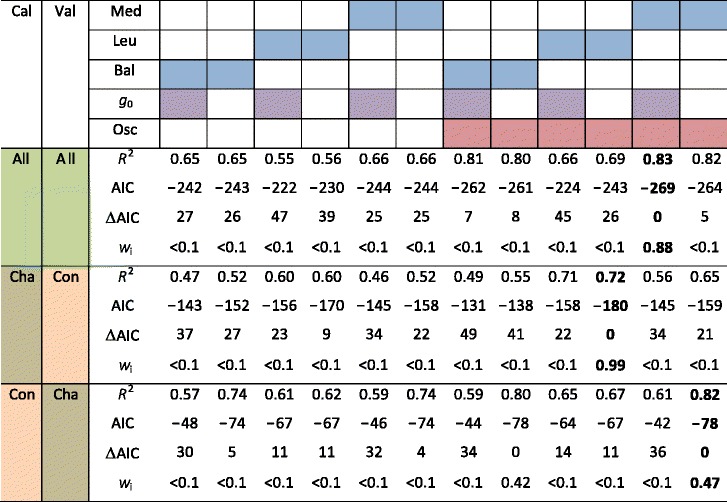
Model fits of leaf stomatal conductance improve when a circadian oscillator is included

Results of fitting the stomatal conductance models proposed by [28] (Med), [29] (Leu), [30] (Bal, indicated in blue), excluding and including minimal conductance (*g*_0_, in purple, a fitting parameter across models, see Analyses), and excluding and including a circadian oscillator (Osc, in red). Data used for calibration (Cal) and validation (Val) are indicated by the colors green (entire data set from Fig. 1b, All), brown (under changing conditions, last 48 h in Fig. 1b, Cha), or orange (under constant conditions, first 24 h in Fig. 1d, Con). Values in bold indicate the best-fit model for each combination of calibration/validation data sets. Models were assessed by *R* ^2^, the Akaike Information Criterion (AIC), AIC reduction (∆AIC) and the weight of each model (*w*_i_). The model with the smallest ∆AIC and largest *w*_i_ is considered the most plausible [32]. Regardless of the data set, inclusion of a circadian oscillator rendered the models more plausible

## Discussion

We observed that, in the absence of fluctuations in environmental drivers, both *A* and *E* statistically significant oscillations through time. According to conventional wisdom, diurnal variation during the entrainment phases is largely attributed to direct environmental effects of PAR, *T*_air_ and VPD on physiological processes [5, 6, 33–35]. However, our experiment using constant environmental conditions as a ‘control’ indicates that 20–79 % of the diurnal range in canopy CO_2_ and H_2_O fluxes can be recreated fully independently of environmental change (Fig. 1, Table 1). There are myriad endogenous processes that could affect diurnal carbon and water flux patterns, including carbohydrate accumulation [36] or hydraulic feedbacks [3]. However, these feedbacks generally tend towards monotonically decreasing *A* and *E* over time. Instead, we observed that diurnal variation under a constant environment showed a period of ~24 h, which is consistent with a circadian gas exchange regulation [12, 37]. Furthermore, we observed that current stomatal models have a high predictive power, but the addition of circadian oscillators led to significant improvements in modeling outputs.

### Circadian regulation of gas exchange from leaves to canopies

It is well known that radiation is the major environmental driver of gas exchange, and can create 100 % of the diurnal oscillation. *T*_air_ and VPD are often considered to be the next most important environmental drivers of diurnal flux dynamics. Although we did not measure the response of *A* or *E* to either *T*_air_ or VPD during these experiments, other studies with these species typically document that, in the absence of strong environmental stress, *T*_air_ and VPD lead to diurnal flux variation of the same order of magnitude as those observed in this study [38]. In other words, the circadian oscillation in *A*_c_ and *E*_c_ observed in this study (Table 1) could be comparable to that documented in *T*_air_ or VPD response curves.

To fully understand the upscaling of circadian rhythms, further exploration is required into how canopy structure determines the canopy-level expression of circadian regulation. Circadian regulation in understory species has been shown to be less important than in overstory species [19]. We hypothesize that this might occur because the predictability and variability of environmental cues diminish under a canopy. We therefore expect a higher dilution of circadian regulation across scales in forests with highly structured multilayer canopies, where a relatively larger proportion of carbon fixation and water loss may be conducted by shaded leaves, than in forests or grasslands with a single layer canopy. This hypothesis could explain why we always observe a higher degree of circadian-driven variation at the leaf-level compared to canopy-level fluxes (Table 1). Additionally, Aschoff’s rules would predict a lengthening of circadian periodicity under low irradiances, which would additionally contribute towards the apparent decrease in amplitude of the circadian rhythm when integrated over the whole canopy [39]. Indeed, there was a higher dilution of circadian regulation at the canopy-level in bean compared to cotton; i.e., the difference between the range of the circadian-driven oscillation between *A*_l_ and *A*_c_, and also between *g*_s_ and *E*_c_, is always higher in bean than in cotton (Table 1). This could be explained by our measurements of a higher LAI, and therefore a higher proportion of shaded leaves, in bean (7.5 m^2^ m^−2^) than in cotton (4.5 m^2^ m^−2^). By measuring only leaf-level gas exchange in the light-exposed upper part of the canopy, we did not capture the reduced rhythms in carbon fixation in the shaded lower part of the canopy; hence the observed dilution across scales is likely the result of not documenting gas exchange in these shaded leaves. Greater understanding of the relative importance of circadian regulation on ecosystem processes, as a function of leaf canopy structure, should be a future research objective. Yet, it is remarkable that despite high LAI in our study, circadian regulation was still apparent at the canopy level.

### Modeling circadian regulation of gas exchange

Similar to previous approaches [22], we conducted a modeling exercise where, among others, *g*_s_ was calibrated with the constant conditions data set, and then validated under changing conditions. Although validation did not occur under strictly field conditions, they were field-like. Hence, the significant improvements in model fits observed when including a circadian oscillator, lead us to conclude that the assertion of circadian rhythms having insignificant rhythms for gas exchange under field settings needs to be revised.

Circadian regulation had a more important effect on stomatal conductance and ecosystem transpiration than on leaf and canopy carbon assimilation (Fig. 1). This may explain why circadian regulation significantly improved stomatal model output in our study, but not in previous studies on photosynthesis [22]. It is worth noting the many reports of hysteresis in tree transpiration in which, for a given environmental condition, transpiration was found to be higher in the morning than in the afternoon [40–42]. This phenomenon has often been explained in terms of hydraulic feedbacks on stomata. However, our results, along with further experiments on circadian regulation of stomata [8, 43], indicate that circadian rhythmicity could at least partly explain hysteretic water fluxes.

Our empirical approach considers time as a surrogate of circadian regulation. Importantly, we observed that the circadian oscillator enhanced the performance of diurnal leaf-level stomatal models (Table 2). We acknowledge that the use of time as a surrogate for circadian action is not fully satisfactory; yet, given present limited understanding of circadian processes at the scale of relevance for this analysis, this is the only approach available.

Previous studies have shown the clock to regulate *g*_s_ independently from *A*_l_ [44, 45]; i.e., the circadian pattern in leaf carbon assimilation is a function of circadian regulation of leaf biochemistry, and independent of variation in stomatal conductance [19, 44, 46]. In this study, our goal was not to assess the mechanisms driving circadian rhythms in stomata and photosynthesis. However, we note that mechanisms underlying circadian gas exchange regulation are mostly studied at molecular or cellular scales. Focusing on the mechanisms underlying circadian regulation, at the scales relevant for ecosystem studies, should be at the forefront of future research efforts.

## Potential implications

To determine the impact of circadian regulation on gas exchange, additional studies across phylogenies and functional groups are required. Although current evidence suggests a highly conserved genetic make-up of circadian rhythm in plants [47], the suite of environmental conditions interacting with circadian regulation of gas exchange remains unknown [19]. Similarly, although our study was performed at much higher PAR levels than in growth chambers (usually < 200 μmol m^−2^ s^−1^), where the circadian clock is most often assessed, PAR was less than full sun and below photosynthetic saturation levels. Additional experiments should address the role of circadian regulation under saturating radiation.

Our results contribute to the expanding field of plant ‘memory’, in that the circadian clock regulates gas exchange based upon the conditions of the previous days. Conceptual frameworks on the effects of ‘memory’ on ecological systems often consider the effect of legacies from antecedent environmental stress [48], and potential epigenetic regulation [49]. Circadian regulation could act as an adaptive memory whereby a plant’s metabolism is adjusted based on the conditions experienced in previous days, and fitness is increased via anticipation [50] and growth regulation [51, 52]. Additional studies are required to expand current frameworks on how to incorporate memory from ecological processes into global change models.

## Methods

The experiment was performed at the Macrocosms platform of the Montpellier European Ecotron, Centre National de la Recherche Scientifique (CNRS, France). Twelve outdoor macrocosms (six planted with bean and six with cotton) were used, where the main abiotic drivers (air temperature, humidity and CO_2_ concentration) were automatically controlled. In each macrocosm, plants were grown on soil (area of 2 m^2^, depth of 2 m) contained in a lysimeter resting on a weighing platform. Soil was collected from the flood plain of the Saale River near Jena, Germany, and used in a previous Ecotron experiment on biodiversity [25]. After that experiment, the soil was ploughed down to 40 cm and fertilized with 25/25/35 NPK (MgO, SO_3_ and other oligo-elements were associated in this fertilizer: Engrais bleu universel, BINOR, Fleury-les-Aubrais, FR).

The soil was regularly watered to *ca* field capacity by drip irrigation, although irrigation was stopped during each measurement campaign (every few days) to avoid interference with water flux measurements. However, no significant differences (at *P* < 0.05, paired *t*-test, *n* = 3) in leaf water potential occurred between the beginning and end of these measurement campaigns, indicating no effect of a potentially declining soil moisture on leaf hydration [21].

Environmental conditions within the macrocosms (excluding the experimental periods) were set to mimic outdoor conditions, but did include a 10 % light reduction by the macrocosm dome cover. During experimental periods, light was controlled by placing a completely opaque fitted cover on each dome to block external light inputs (polyvinyl chloride-coated polyester sheet Ferrari 502, assembled by IASO, Lleida, Spain), and by using a set of five dimmable plasma lamps (GAN 300 LEP with the Luxim STA 4102 bulb, with a sun-like light spectrum, see Additional file 2); these lamps were hung 30 cm above the plant canopy and provided a PAR of 500 μmol m^−2^ s^−1^ (the maximum possible with these lamps). PAR was measured at the canopy level with a quantum sensor (Li-190, LI-COR Biosciences, Lincoln, NE, USA) in each macrocosm and corroborated a continuous PAR of 500 μmol m^−2^ s^−1^ during the experiment.

Bean and cotton were planted in five different rows within the domes on 10^th^ July 2013, one month before the start of the measurements, and thinned to densities of 105 and 9 individuals per square meter, respectively. Cotton (STAM-A16 variety, Institut National de Recherche Agronomique du Bénin (INRAB) / Centre de Coopération Internationale en Recherche Agronomique pour le Développement (CIRAD)) is a perennial shrub with an indeterminate growth habit. This cotton variety grows to 1.5–2 m tall and has a pyramidal shape and short branches. Bean is an annual herbaceous species. Recombinant inbred line (RIL)-115 (bred by the Institut National de la Recherche Agronomique (INRA) ‘Eco & Sols’) was used, which is a fast growing, indeterminate dwarf variety, growing 0.3–0.5 m tall. Bean plants were inoculated with *Rhizobium tropici* CIAT 899, also provided by INRA. During the experiment, bean and cotton generally remained at the inflorescence emergence developmental growth stage ([53], codes 51–59 in the BBCH scale, the standard phenological scale within the crop industry; [54]). No specific license or permission was required.

## Additional files

Additional file 1: Further details on stomatal models. (PDF 25 kb)
Additional file 2: The plasma lamps used in the experiment had a sun-light spectrum. Intensity at each wavelength was measured with a Jaz spectrometer (Ocean Optics UV-NIR detector, Jasper, GA, USA). (PDF 187 kb)

## Abbreviations

*A*_c_: Canopy net CO_2_ exchange
AIC: Akaike information criterion
*A*_l_: Leaf level net photosynthesis
CIRAD: Centre de Coopération Internationale en Recherche Agronomique pur le Développement (Center for International Cooperation in Agronomic Research for Development)
CNRS: Centre National de la Recherche Scientifique (French National Center for Scientific Research)
*E*_c_: Canopy transpiration
GAMM: Generalized additive mixed model
*g*_s_: Stomatal conductance
INRA: Institut National de la Recherche Agronomique (French National Institute for Agronomic Research)
INRAB: Institut National de la Recherche Agronomique du Bénin (Benin National Institute for Agronomic Research)
LAI: Leaf area index
PAR: Photosynthetically active radiation
RIL: Recombinant inbred line
*T*_air_: Air temperature
VPD: Vapor pressure deficit.

## References

[1] Canadell JG, Le Quere C, Raupach MR, Field CB, Buitenhuis ET, Ciais P (2007). Contributions to accelerating atmospheric CO_2_ growth from economic activity, carbon intensity, and efficiency of natural sinks. Proc Natl Acad Sci U S A..

[2] Schlesinger WH, Jasechko S (2014). Transpiration in the global water cycle. Agr Forest Meteorol..

[3] Jones H (1998). Stomatal control of photosynthesis and transpiration. J Exp Bot..

[4] Chapin FS, Matson PA, Mooney HA (2002). Principles of Terrestrial Ecosystem Ecology.

[5] Sellers PJ, Dickinson RE, Randall DA, Betts AK, Hall FG, Berry JA (1997). Modeling the exchanges of energy, water, and carbon between continents and the atmosphere. Science..

[6] Hollinger DY, Kelliher FM, Byers JN, Hunt JE, McSeveny TM, Weir PL (1994). Carbon dioxide exchange between an undisturbed old-growth temperate forest and the atmosphere. Ecology..

[7] Resco V, Hartwell J, Hall A (2009). Ecological implications of plants' ability to tell the time. Ecol Lett..

[8] Mencuccini M, Mambelli S, Comstock J (2000). Stomatal responsiveness to leaf water status in common bean (Phaseolus vulgaris L.) is a function of time of day. Plant Cell Environ.

[9] Hubbard KE, Webb AAR, Mancuso S, Shabala S (2015). Circadian rhythms in stomata: Physiological and molecular aspects. Rhythms in Plants.

[10] Paul MJ, Pellny TK (2003). Carbon metabolite feedback regulation of leaf photosynthesis and development. J Exp Bot..

[11] Webb AAR (2003). The physiology of circadian rhythms in plants. New Phytol..

[12] Salmela MJ, Greenham K, Lou P, McClung CR, Ewers BE, Weinig C (2016). Variation in circadian rhythms is maintained among and within populations in Boechera stricta. Plant Cell Environ..

[13] Ehleringer JR, Field CB (1993). Scaling physiological processes.

[14] Lasslop G, Reichstein M, Papale D, Richardson AD, Arneth A, Barr A (2010). Separation of net ecosystem exchange into assimilation and respiration using a light response curve approach: critical issues and global evaluation. Glob Change Biol..

[15] Williams M, Rastetter EB, Van der Pol L, Shaver GR (2014). Arctic canopy photosynthetic efficiency enhanced under diffuse light, linked to a reduction in the fraction of the canopy in deep shade. New Phytol..

[16] Dietze MC (2014). Gaps in knowledge and data driving uncertainty in models of photosynthesis. Photosynth Res..

[17] Stoy PC, Trowbridge AM, Bauerle WL (2014). Controls on seasonal patterns of maximum ecosystem carbon uptake and canopy-scale photosynthetic light response: contributions from both temperature and photoperiod. Photosynth Res..

[18] Resco de Dios V, Goulden ML, Ogle K, Richardson AD, Hollinger DY, Davidson EA (2012). Endogenous circadian regulation of carbon dioxide exchange in terrestrial ecosystems. Glob Change Biol.

[19] Doughty C, Goulden ML, Miller S, da Rocha H (2006). Circadian rhythms constrain leaf and canopy gas exchange in an Amazonian forest. Geophys Res Lett..

[20] Resco de Dios V, Diaz-Sierra R, Goulden ML, Barton CV, Boer MM, Gessler A (2013). Woody clockworks: circadian regulation of night-time water use in Eucalyptus globulus. New Phytol.

[21] Resco de Dios V, Roy J, Ferrio JP, Alday JG, Landais D, Milcu A (2015). Processes driving nocturnal transpiration and implications for estimating land evapotranspiration. Sci Rep.

[22] Williams WE, Gorton HL (1998). Circadian rhythms have insignificant effects on plant gas exchange under field conditions. Physiol Plantarum..

[23] Roy J, Picon-Cochard C, Augusti A, Benot M-L, Thiery L, Darsonville O (2016). Elevated CO_2_ maintains grassland net carbon uptake under a future heat and drought extreme. Proc Natl Acad Sci U S A..

[24] Wood SN (2006). Generalized Additive Models: An Introduction. R.

[25] Milcu A, Roscher C, Gessler A, Bachmann D, Gockele A, Guderle M (2014). Functional diversity of leaf nitrogen concentrations drives grassland carbon fluxes. Ecol Lett..

[26] Pinheiro JC, Bates DM (2000). Mixed-Effects Models in S and S-PLUS.

[27] Curtis CJ, Simpson GL (2014). Trends in bulk deposition of acidity in the UK, 1988–2007, assessed using additive models. Ecol Indic..

[28] Medlyn BE, Duursma RA, Eamus D, Ellsworth DS, Prentice IC, Barton CVM, et al. Reconciling the optimal and empirical approaches to modelling stomatal conductance. Glob Change Biol;2011;17:2134–2144.

[29] Leuning R (1995). A critical appraisal of a combined stomatal-photosynthesis model for C3 plants. Plant Cell Environ..

[30] Ball TJ, Woodrow IE, Berry JA, Biggins J (1987). A model predicting stomatal conductance and its contribution to the control of photosynthesis under different environmental conditions. Progress in Photosynthesis Research.

[31] Wagenmakers E-J, Farrell S (2004). AIC model selection using Akaike weights. Psychon Bull Rev..

[32] Burnham KP, Anderson DR (2002). Model Selection and Multi/Model Inference: A Practical Information-Theoretic Approach.

[33] Richardson AD, Hollinger DY, Aber JD, Ollinger SV, Braswell BH (2007). Environmental variation is directly responsible for short- but not long-term variation in forest-atmosphere carbon exchange. Glob Change Biol..

[34] Jones H (2014). Plants and microclimate: a quantitative approach to environmental plant physiology.

[35] Schwalm CR, Williams CA, Schaefer K, Anderson R, Arain MA, Baker I, et al. A model-data intercomparison of CO_2_ exchange across North America: Results from the North American Carbon Program site synthesis. J Geophys Res. 2010;115:G00H05.

[36] Azcón-Bieto J (1983). Inhibition of photosynthesis by carbohydrates in wheat leaves. Plant Physiol..

[37] Müller LM, von Korff M, Davis SJ (2014). Connections between circadian clocks and carbon metabolism reveal species-specific effects on growth control. J Exp Bot..

[38] Duursma RA, Barton CVM, Lin Y-S, Medlyn BE, Eamus D, Tissue DT (2014). The peaked response of transpiration rate to vapour pressure deficit in field conditions can be explained by the temperature optimum of photosynthesis. Agr Forest Meteorol..

[39] Beaulé C, Binder MD, Hirokawa N, Windhorst U (2009). Aschoff's Rules. Encyclopedia of Neuroscience.

[40] Zhang Q, Manzoni S, Katul G, Porporato A, Yang D (2014). The hysteretic evapotranspiration—Vapor pressure deficit relation. J Geophys Res.

[41] Tuzet A, Perrier A, Leuning R (2003). A coupled model of stomatal conductance, photosynthesis and transpiration. Plant Cell Environ.

[42] O'Grady AP, Eamus D, Hutley LB (1999). Transpiration increases during the dry season: Patterns of tree water use in eucalypt open-forests of northern Australia. Tree Physiol..

[43] Marenco RA, Siebke K, Farquhar GD, Ball MC (2006). Hydraulically based stomatal oscillations and stomatal patchiness in Gossypium hirsutum. Funct Plant Biol..

[44] Dodd AN, Kusakina J, Hall A, Gould PD, Hanaoka M (2014). The circadian regulation of photosynthesis. Photosynth Res..

[45] Dodd AN, Parkinson K, Webb AAR (2004). Independent circadian regulation of assimilation and stomatal conductance in the ztl-1 mutant of *Arabidopsis*. New Phytol..

[46] Haydon MJ, Mielczarek O, Robertson FC, Hubbard KE, Webb AA (2013). Photosynthetic entrainment of the Arabidopsis thaliana circadian clock. Nature..

[47] Holm K, Källman T, Gyllenstrand N, Hedman H, Lagercrantz U (2010). Does the core circadian clock in the moss *Physcomitrella patens* (Bryophyta) comprise a single loop?. BMC Plant Biol..

[48] Ogle K, Barber JJ, Barron-Gafford GA, Bentley LP, Young JM, Huxman TE (2015). Quantifying ecological memory in plant and ecosystem processes. Ecol Lett..

[49] Crisp PA, Ganguly D, Eichten SR, Borevitz JO, Pogson BJ (2016). Reconsidering plant memory: Intersections between stress recovery, RNA turnover, and epigenetics. Sci Adv..

[50] Resco De Dios V, Loik ME, Smith RA, Aspinwall MJ, Tissue DT (2016). Genetic variation in circadian regulation of nocturnal stomatal conductance enhances plant fitness. Plant Cell Environ.

[51] Herrmann S, Recht S, Boenn M, Feldhahn L, Angay O, Fleischmann F (2015). Endogenous rhythmic growth in oak trees is regulated by internal clocks rather than resource availability. J Exp Bot..

[52] Graf A, Schlereth A, Stitt M, Smith AM (2010). Circadian control of carbohydrate availability for growth in Arabidopsis plants at night. Proc Natl Acad Sci USA.

[53] Munger L, Bleiholder H, Hack H, Hess M, Stauss R, Boom TVD (1998). Phenological growth stages of the peanut plant (Arachis hypogaea L.) Codification and description according to the BBCH Scale – with figures. J Agron Crop Sci.

[54] Feller C, Bleiholder H, Buhr L, Hack H, Hess M, Klose R (1995). Phänologische Entwicklungsstadien von Gemüsepflanzen: II. Fruchtgemüse und Hülsenfrüchte. Nachrichtenbl Deut. Pflanzenschutzd.

[55] Resco de Dios V, Gessler A, Ferrio JP, Alday JG, Bahn M, del Castillo J, et al. Supporting data for "Circadian rhythms have significant effects on leaf-to-canopy scale gas exchange under field conditions". 2016. GigaScience Database http://dx.doi.org/10.5524/100244 10.1186/s13742-016-0149-y PMC5072338 27765071

[56] Hennessey T, Freeden A, Field C (1993). Environmental effects on circadian rhythms in photosynthesis and stomatal opening. Planta..

