# Supplementary figures and images for: Circadian rhythms have significant effects on leaf-to-canopy scale gas exchange under field conditions
Source: Gigascience. 2016 Oct 20;5:43. doi: 10.1186/s13742-016-0149-y (PMC5072338; doi:10.1186/s13742-016-0149-y)

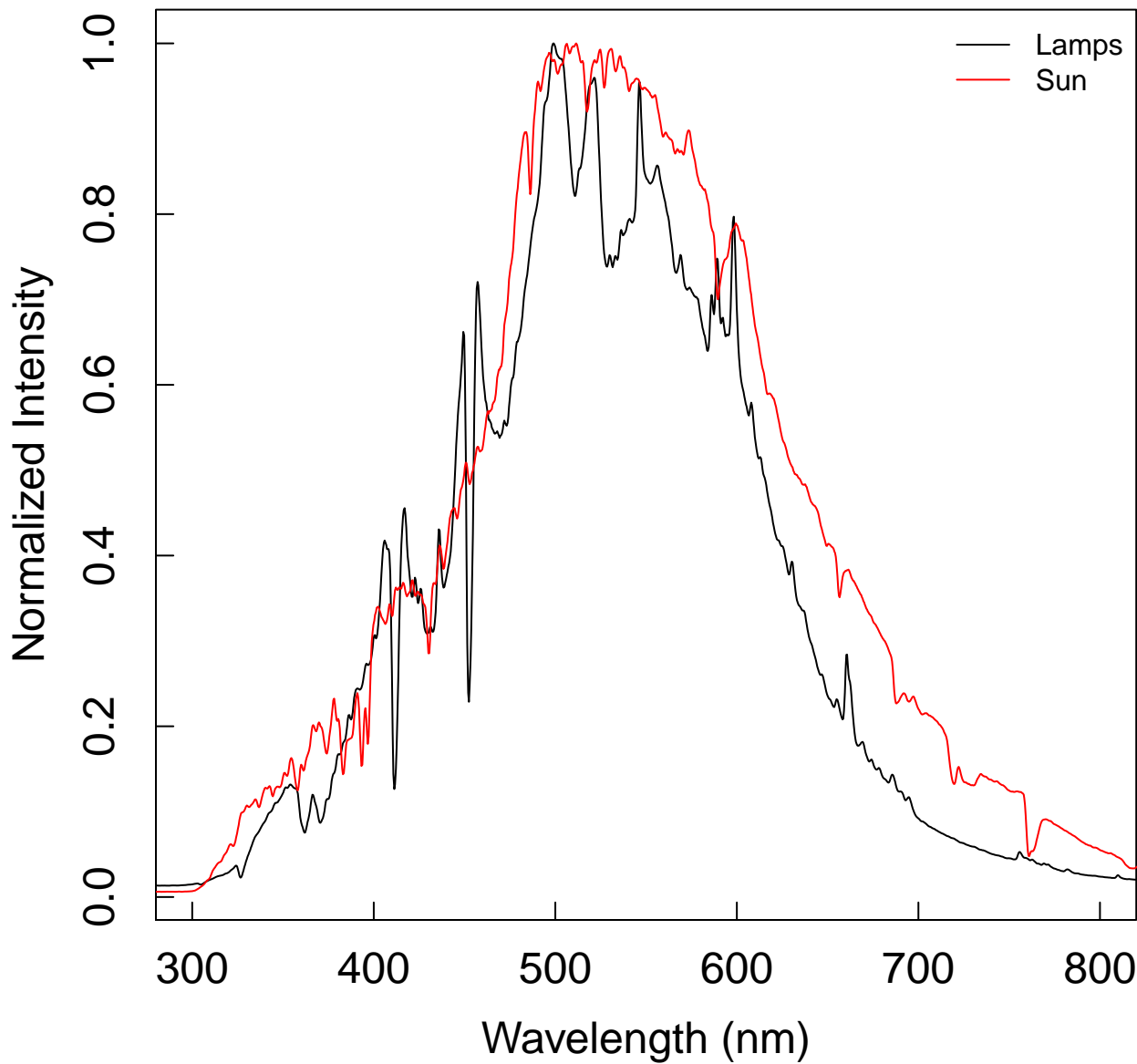

Supplement: Additional file 1: — Further details on stomatal models. (PDF 25 kb) [file 13742_2016_149_MOESM1_ESM.pdf]
