# Supplementary material for: Circadian rhythms have significant effects on leaf-to-canopy scale gas exchange under field conditions
Source: Gigascience. 2016 Oct 20;5:43. doi: 10.1186/s13742-016-0149-y (PMC5072338; doi:10.1186/s13742-016-0149-y)
Supplement: Additional file 2: — The plasma lamps used in the experiment had a sun-light spectrum. Intensity at each wavelength was measured with a Jaz spectrometer (Ocean Optics UV-NIR detector, Jasper, GA, USA). (PDF 187 kb) [file 13742_2016_149_MOESM2_ESM.pdf]

A commonality across the stomatal models used [1-3] is that photosynthesis (A_l) acts as a driver of stomatal conductance (g_s). We will now describe the model of Medlyn et al. [1], but we note that the same rationale applies to all other models. There are three variables driving this model:

$$g_s = g_0 + \left(1 + \frac{g_1}{\sqrt{VPD}}\right)\frac{A_l}{C_a}$$

namely, vapor pressure deficit (VPD), A_l and CO_2 concentration (C_a), with two fitting parameters (g_0 and g_1). Given that g_s and A_l are usually correlated [4, 5], even under constant environmental conditions the model has a reasonable goodness-of-fit (see Table 2), which is further enhanced by the addition of circadian oscillators (Table 2).

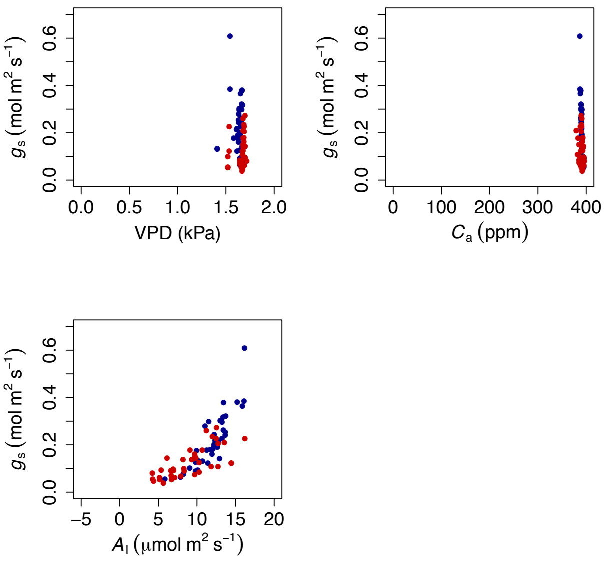

1. Medlyn BE, Duursma RA, Eamus D, Ellsworth DS, Prentice IC, Barton CVM, Crous KY, De Angelis P, Freeman M, Wingate L. Reconciling the optimal and empirical approaches to modelling stomatal conductance. Glob Change Biol. 2011;17:2134-2144.
2. Ball TJ, Woodrow IE, Berry JA. A model predicting stomatal conductance and its contribution to the control of photosynthesis under different environmental conditions. In: Biggins J, editor. Progress in Photosynthesis Research. vol. 4. Dordrecht: Martinus Nijhoff;1987:221-224.
3. Leuning R. A critical appraisal of a combined stomatal-photosynthesis model for C3 plants. Plant Cell Environ 1995;18:339-355.
4. Cowan IR, Farquhar GD. Stomatal function in relation to leaf metabolism and environment. In: Jennings DH, editor. Integration of Activity in the Higher Plant. Cambridge: Cambridge University Pres; 1977.
5. Wong SC, Cowan IR, Farquhar GD. Stomatal conductance correlates with photosynthetic capacity. Nature 1979;282:424-426.
